# Association of estimated glomerular filtration rate and incident pre-diabetes: A secondary 5-year longitudinal cohort study in Chinese people

**DOI:** 10.3389/fendo.2022.965545

**Published:** 2022-10-27

**Authors:** Xiaoyu Wang, Cheng Huang, Yufei Liu, Yong Han, Haofei Hu

**Affiliations:** ^1^ Department of Nephrology, Hechi People’s Hospital, Hechi, Guangxi Zhuang Autonomous Region, China; ^2^ Department of Neurosurgery, Shenzhen Second People’s Hospital, Shenzhen, Guangdong, China; ^3^ Department of Neurosurgery, The First Affiliated Hospital of Shenzhen University, Shenzhen, Guangdong, China; ^4^ Shenzhen University Health Science Center, Shenzhen University, Shenzhen, Guangdong, China; ^5^ Department of Emergency, Shenzhen Second People’s Hospital, Shenzhen, Guangdong, China; ^6^ Department of Emergency, The First Affiliated Hospital of Shenzhen University, Shenzhen, Guangdong, China; ^7^ Department of Nephrology, Shenzhen Second People’s Hospital, Shenzhen, Guangdong, China; ^8^ Department of Nephrology, The First Affiliated Hospital of Shenzhen University, Shenzhen, Guangdong, China

**Keywords:** prediabetes, evaluated glomerular filtration rate, cohort study, U-shaped curve relationship, Cox proportional-hazards regression

## Abstract

**Objective:**

There is still limited evidence regarding the relationship between the estimated glomerular filtration rate (eGFR) and pre-diabetes. For that reason, our research aims to survey the association of eGFR with pre-diabetes.

**Methods:**

This study was a retrospective cohort study, which consecutively and non-selectively collected a total of 173301 participants from Rich Healthcare Group in China from January 2010 to 2016. We then used the Cox proportional-hazards regression model to explore the relationship between baseline eGFR and pre-diabetes risk. Using a Cox proportional hazards regression with cubic spline function and smooth curve fitting (cubical spline smoothing), we were able to determine the non-linear relationship between eGFR and pre-diabetes. Additionally, we also conducted a series of sensitivity analyses and subgroup analyses. The DATADRYAD website was updated with data.

**Results:**

The mean age of the included individuals was 40.95 ± 11.94 years old, and 92318 (53.27%) were male. The mean baseline eGFR was 111.40 ± 14.77 ml/min per 1.73 m^2^. During a median follow-up time of 3.0 years, 18333 (10.58%) people experienced pre-diabetes. As a result of adjusting for covariates, eGFR had a negative association with incident pre-diabetes (HR=0.993, 95%CI: 0.992-0.995). There was also a U-shaped curve relationship between eGFR and pre-diabetes, and the inflection point of eGFR was 129.793 ml/min per 1.73 m^2^. HRs on the left and right sides of the inflection point were respectively 0.993 (0.991-0.994) and 1.023 (1.010- 1.037). Our results were robust in the sensitivity analysis. Subgroup analyses indicated that eGFR was strongly associated with the risk of pre-diabetes among participants who were younger than 30 years and 40-70 years, as well as among those who had never smoked. In contrast, the association of eGFR with the risk of pre-diabetes was attenuated among participants who were 30-40 years of age and 70 years of age or older, and among those who currently smoked.

**Conclusion:**

This study demonstrates a negative and U-shaped curve association between eGFR and the risk of pre-diabetes among the general Chinese population. Either reduced renal function or glomerular hyperperfusion status may be associated with an increased risk of prediabetes.

## Background

A state of hyperglycemia known as pre-diabetes is one in which blood sugar levels are higher than normal, but lower than those of diabetics (1). According to 2013 estimates, the prevalence of pre-diabetes among Chinese adults was 35.7% (2). Approximately 5-10% of prediabetic patients progress to diabetes mellitus (DM) every year, and 70% of them develop DM eventually (3). Prediabetic individuals are at an increased risk of a variety of complications of diabetes in the future, including macrovascular complications (for example, cardiovascular disease) and microvascular complications (such as kidney, retina, and nervous system complications) (4–6). Furthermore, the hyperglycemia status before the onset of diabetes deteriorates the kidney, nervous system, retina, and macro-vessels (7–9). The burden of prediabetic-related diseases and disorders has weighed heavily on families and society. Therefore, pre-diabetes is a risk factor for developing diabetes and its complications, so identifying and treating such individuals is critical.

There are currently no unified criteria for diagnosing pre-diabetes, which is characterized as impaired glucose tolerance or impaired fasting glucose (IFG) (10). Pre-diabetes diagnostic criteria defined by the American Diabetes Association (ADA) are widely used in China. Thus, fasting plasma glucose (FPG):5.6–6.9 mmol/L is defined as the IFG threshold (11).

Diagnosing chronic kidney disease (CKD) is done using the estimated glomerular filtration rate (eGFR), which is a more accurate and direct indicator of the renal filtration function (12). Several previous population-based studies were not able to find any association of pre-diabetes with CKD nor with decreased GFR after adjusting for risk factors (13). However, a recent cohort study showed an overall trend towards a slightly decreased risk of impaired kidney function onset associated to pre-diabetes with an adjusted hazard ratio (HR) of 0.76. However, this finding is restricted to subjects who only had impaired glycated hemoglobin (HbA1c) and impaired fasting plasma glucose; instead, subjects with only impaired FPG levels had a slightly increased risk of reduced kidney function (14). A recent meta-analysis, including eight cohort studies with subjects with impaired FPG as pre-diabetes criteria, has also reported a modestly increased risk of impaired renal function associated with impaired FPG (15). A Chinese study found that FPG, but not 2-h postload blood glucose or HbA1c, is associated with a mild decline in eGFR in healthy Chinese people (16). Another Chinese study found an increased risk of glomerular hyperfiltration in patients with impaired glucose tolerance and newly diagnosed diabetes (17). Our previous study demonstrated an inverse and non-linear relationship between eGFR and the risk of diabetes in a community-based population in China (18). However, reviewing the literature, we found no evidence of the relationship between eGFR (both lower eGFR and higher eGFR) and pre-diabetes. Therefore, we performed a cohort study to investigate the relationship between eGFR and pre-diabetes risk in the Chinese community.

## Methods

### Study design

We performed a retrospective cohort study using the data from the database provided by China Rich Healthcare Group. The eGFR at baseline was the interesting independent variable in the present study. The dependent variable was prediabetes diagnosed during follow-up (dichotomous variable: 0 = non-prediabetes, 1= prediabetes).

### Data source

The raw data was taken from the DATADRYAD database (www.datadryad.org) for free provided by Chen, Ying et al. (2018), Data from: Association of body mass index and age with incident diabetes in Chinese adults: a population-based cohort study, Dryad, Dataset, https://doi.org/10.5061/dryad.ft8750v. Under Dryad’s terms of service, researchers could use this data for secondary analyses without violating authors’ rights.

### Study population

Because the most frequent bias was selection bias, which could lead to an over/underestimation of the obtained results. For the purpose of minimizing selection bias, participants who underwent a health examination were collected non-selectively and consecutively from 32 locations in 11 cities in China (Beijing, Suzhou, Nanjing, Shanghai, Changzhou, Shenzhen, Nantong, Chengdu, Hefei, Guangzhou, and Wuhan). Their identity information was encoded as non-traceable codes to ensure participants’ privacy. The data were extracted from a computerized database established by the Rich Healthcare Group in China, which included all medical records for participants who received a health check from 2010 to 2016. The Rich Healthcare Group Review Board approved the original study, and the informed consent was waived because of the retrospective nature of this study (19).

**Figure 1:**
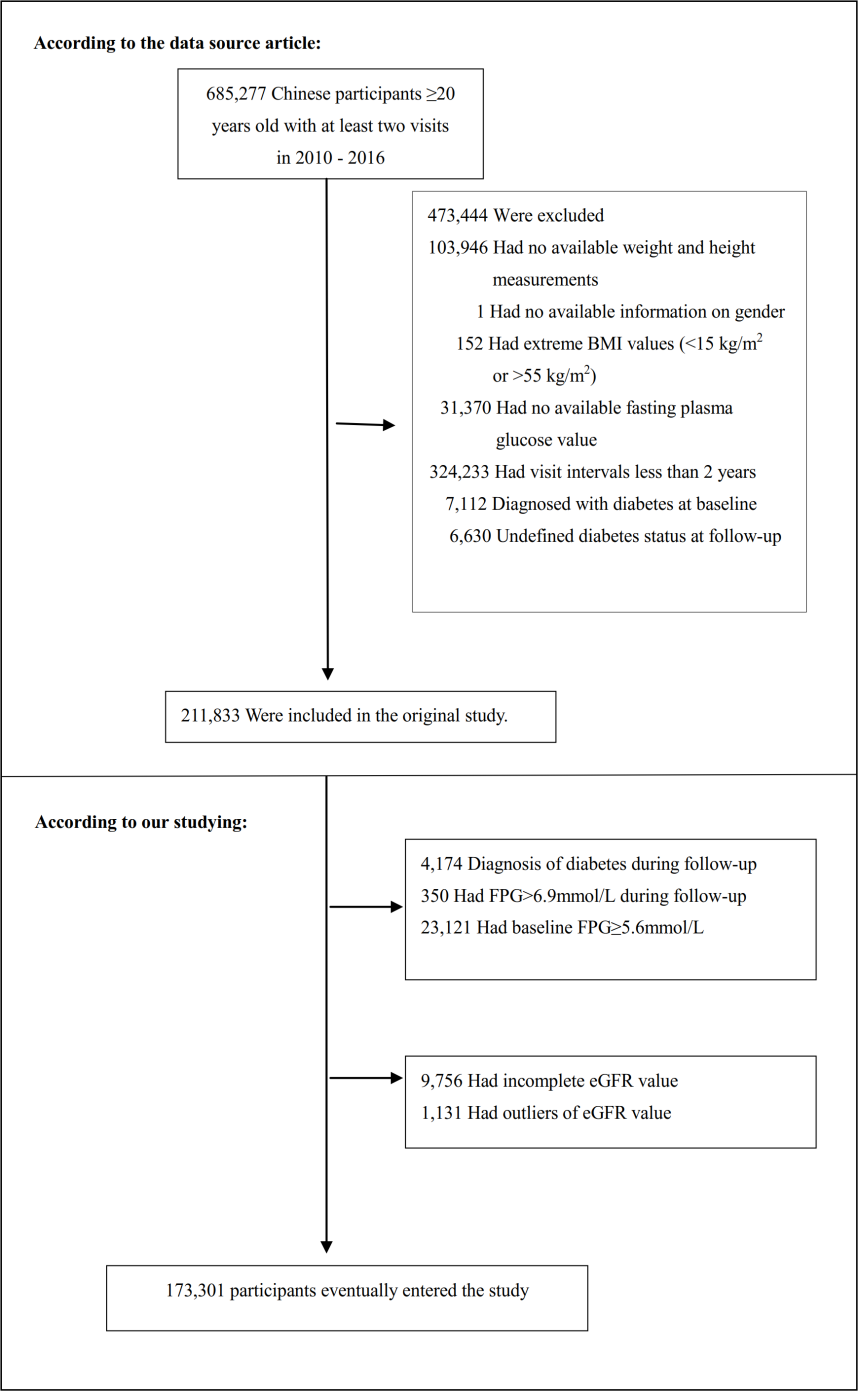
Flowchart of study participants. Figure 1 showed the inclusion of participants. 211833 participants were assessed for eligibility in the original study. We further excluded 38532 participants. The final analysis included 173301 subjects in the present study.

The study initially collected 685277 participants; afterward, 511976 participants were excluded, and 173301 participants were left for data analysis (see flowchart for details in **Figure 1**). Those who were at least 20 years old, had a health screening and had at least two visits between 2010 and 2016 were included in the study. Exclusion criteria included (20): (1) participants with no available information about baseline fasting blood glucose (FPG), weight, gender, or height (n=135317); (2) participants whose visit period was less than 2 years (n=324233); (3) those with extreme BMI values (<15 kg/m^2^ or >55 kg/m^2^) (n=152); (4) participants with unknown diabetes status at follow-up (n=6630); (5) those diagnosed with diabetes at baseline (n=7112); (6) participants with self-reported DM or FPG≥ 6.9 mmol/L in the follow-up period (n=4524); (7) participants with baseline FPG≥5.6 mmol/L (n=23121); (8) participants with incomplete eGFR (n=9756); (9) those with eGFR outliers(out of the range of means plus or minus three standard deviations) (n=1131) (21, 22). Compared with individuals excluded from the original study, those included in the original analyses were with similar age (42.1 vs. 41.9 years old) and similar BMI (23.2 vs. 23.3 kg/m2), and a similar proportion of males (54.8% vs. 52.1%). Compared to the original study, this study excluded only a smaller proportion of participants (5.6%).

### Variables

#### Estimated glomerular filtration rate

We obtained the information on eGFR at baseline and recorded it as a continuous variable. Chronic Kidney Disease Epidemiology Collaboration (CKD-EPI) equations were used to calculate the eGFR for “Asian origin” patients (23). It was calculated based on gender, age, and serum creatinine (Scr) with the following formula:

Females with the concentration of Scr ≤0.7 mg/dL, eGFR = 151 × (Scr/0.7)^-0.328^ ×0.993^age^; Females with the concentration of Scr >0.7 mg/dL, eGFR = 151 × (Scr/0.7)^-1.210^ ×0.993^age^; Males with the concentration of Scr ≤ 0.9 mg/dL, eGFR = 149 × (Scr/0.9)^-0.415^ ×0.993^age^; Males with the concentration of Scr >0.9 mg/dL, eGFR = 149 × (Scr/0.9)^-1.210^ ×0.993^age^;

The unit of age and Scr was year and mg/dL, respectively. Based on the Asian modified CKD-EPI equation, Chinese CKD patients’ GFR could be more accurately determined, especially in populations with high GFRs.

#### Outcome measures

Our interesting outcome variable was pre-diabetes (dichotomous variable: 0= non-prediabetes, 1= pre-diabetes). Pre-diabetes was diagnosed based on IFG, and FPG values in prediabetic patients were set at 5.6 to 6.9 mmol/L as part of the ADA’s 2018 diagnostic criteria (11). We censored participants at the time of pre-diabetes diagnosis or the last visit, whichever came first. The follow-up period was 5 years.

#### Covariates

Based on previous research and clinical experience, we chose covariates for our study (20). The following variables were therefore used as covariates based on the principles outlined above: (1) continuous variables: systolic blood pressure (SBP), body mass index (BMI), age, diastolic blood pressure (DBP), triglyceride (TG), FPG, total cholesterol (TC), Blood urea nitrogen (BUN), high-density lipoprotein cholesterol (HDL-c), aspartate aminotransferase (AST), low-density lipoprotein cholesterol (LDL-c), alanine aminotransferase (ALT); (2) categorical variables: smoking status, gender, drinking status, and family history of diabetes.

Every time they visited the health check center, a detailed questionnaire was provided to each participant, including their lifestyle, family history of chronic illness, demographic characteristics, and personal medical history. Weight, blood pressure, and height were measured by trained staff. When measuring weight, wear light clothing and no shoes, and measure with an accuracy of 0.1 kg. Weight (kg) was divided by height (m) squared to determine BMI. Height was measured accurately to within 0.1 cm. Blood pressure was obtained by trained staff using standard mercury sphygmomanometers through office blood pressure measurements. Before taking blood pressure, the examinee should lie down and rest quietly for 5-10 minutes. Smoking status was divided into three categories according to the smoking situation: currently smoking, ever smoking and never smoking. The drinking status was divided into three categories according to the drinking situation: currently drinking, ever drinking and never drinking. Smoking and drinking status were assessed only at baseline. Fasting venous blood samples were collected after fasting for at least 10 hours at each appointment. HDL-c, AST, Scr, TC, BUN, TG, FPG, ALT, and LDL-c were measured on an autoanalyzer (Beckman 5800) (20).

### Statistical analysis

Quartiles of eGFR stratified the participants. In the case of continuous variables, baseline characteristics are presented as mean ± standard deviation (SD) (Gaussian distribution) or median (range) (Skewed distribution), and as percentages for categorical variables. We used three kinds of statistical tests to detect the differences among different eGFR groups: χ2 for categorical variables, One-Way ANOVA for normal distribution, or Kruskal-Whallis H for skewed distribution. To compute the survival estimates and time-to-event variables, we employed the Kaplan-Meier method. We used the log-rank test to compare the probability of prediabetes-free survival among the eGFR groups (24).

In order to assess covariate colinearity, the variance inflation factor (VIF) was calculated (25). VIF = 1/(1-R^2^). Where R^2^ was the R-squared value from a linear regression equation where the dependent variable was this variable, and the independent variables were all other variables. If the VIF was greater than 5, then the variables would be considered collinear and could not be included in the multiple regression model (**Table S1**).

After collinearity screening, we tested three distinct models for the relation between eGFR and pre-diabetes using the univariate and multivariate Cox proportional-hazards regression method. As for model I, it was the nonadjusted model with no covariates adjusted. As for model II, it was the minimally-adjusted model with only sociodemographic variables adjusted, including SBP, gender, DBP, age, family history of diabetes, BMI, drinking and smoking status. Model III was the fully-adjusted model with covariates presented in **Table 1**, including SBP, gender, FPG, age, BMI, BUN, DBP, TG, ALT, HDL-c, AST, family history of diabetes, LDL-c, drinking and smoking status. We recorded the effect sizes (HR) and 95% confidence intervals (CIs) and adjusted them when the covariances were added to the model, and the hazard ratio (HR) changed by 10% or more (26). Also, it referred to the results of the collinearity screening. We found that TC was collinear with other variables (**Table S1**), so we did not finally include TC in the multivariate Cox proportional-hazards regression equation.

**Table 1:** The Baseline Characteristics of Participants.

eGFR group	<101.66	101.66-113.55	113.55-122.69	≥122.69	P-value
**Participants**	43316	43330	43318	43337	
**Age(years)**	51.1 ± 14.2	43.7 ± 10.0	37.4 ± 6.2	31.5 ± 4.4	< 0.001
**BMI(kg/m^2^)**	23.9 ± 3.1	23.4 ± 3.1	22.8 ± 3.2	22.0 ± 3.3	< 0.001
**SBP(mmHg)**	122.8 ± 17.5	118.5 ± 15.5	115.4 ± 14.3	114.0 ± 13.8	< 0.001
**DBP(mmHg)**	76.1 ± 11.0	74.5 ± 10.7	72.5 ± 10.1	70.8 ± 9.6	< 0.001
**FPG(mmol/L)**	4.8 ± 0.5	4.8 ± 0.5	4.7 ± 0.5	4.7 ± 0.5	< 0.001
**TC(mmol/L)**	4.9 ± 0.9	4.8 ± 0.9	4.6 ± 0.8	4.4 ± 0.8	< 0.001
**TG(mmol/L)**	1.2 (0.9-1.8)	1.1 (0.8-1.7)	1.0 (0.7-1.4)	0.8 (0.6-1.2)	< 0.001
**HDL-c(mmol/L)**	1.3 ± 0.3	1.4 ± 0.3	1.4 ± 0.3	1.4 ± 0.3	< 0.001
**LDL-c(mmol/L)**	2.9 ± 0.7	2.8 ± 0.7	2.6 ± 0.6	2.5 ± 0.6	< 0.001
**ALT(U/L)**	19.0 (14.0-27.4)	18.8 (13.3-28.0)	17.0 (12.1-27.0)	15.0 (11.0-23.4)	< 0.001
**AST(U/L)**	23.4 (19.0-29.0)	22.5 (18.0-28.1)	21.6 (17.3-27.3)	20.5 (16.4-26.0)	< 0.001
**eGFR(mL/min·1.73 m^2^)**	91.0 ± 8.4	107.9 ± 3.4	118.3 ± 2.6	128.4 ± 4.5	< 0.001
**Scr(umol/L)**	82.6 ± 13.7	71.4 ± 12.1	65.7 ± 10.9	57.9 ± 10.1	< 0.001
**BUN(mmol/L)**	5.0 ± 1.2	4.7 ± 1.1	4.4 ± 1.1	4.2 ± 1.1	< 0.001
**Gender**					< 0.001
** Male**	28488 (65.8%)	25234 (58.2%)	21802 (50.3%)	16794 (38.8%)	
** Female**	14828 (34.2%)	18096 (41.8%)	21516 (49.7%)	26543 (61.2%)	
**Smoking status**					< 0.001
** Never smoker**	32083 (74.1%)	33539 (77.4%)	35925 (82.9%)	38115 (88.0%)	
** Ever smoker**	1703 (3.9%)	1665 (3.8%)	1666 (3.8%)	1321 (3.0%)	
** Current smoker**	9530 (22.0%)	8126 (18.8%)	5727 (13.2%)	3901 (9.0%)	
**Drinking status**					< 0.001
** Never drinker**	35711 (82.4%)	36648 (84.6%)	37574 (86.7%)	39007 (90.0%)	
** Ever drinker**	6494 (15.0%)	5825 (13.4%)	5196 (12.0%)	4008 (9.2%)	
** Current drinker**	1111 (2.6%)	857 (2.0%)	548 (1.3%)	322 (0.7%)	
**Family history of diabetes**					< 0.001
** No**	42606 (98.4%)	42392 (97.8%)	42285 (97.6%)	42499 (98.1%)	
** Yes**	710 (1.6%)	938 (2.2%)	1033 (2.4%)	838 (1.9%)	

Values are n(%), mean ± SD or medians (quartiles).

BMI, body mass index; FPG, fasting plasma glucose; DBP, diastolic blood pressure; TC, total cholesterol; SBP, systolic blood pressure; TG, triglyceride; ALT, alanine aminotransferase; LDL-c, low-density lipid cholesterol; AST, aspartate aminotransferase; HDL-c, high-density lipoprotein cholesterol; eGFR, estimated glomerular filtration rate; BUN, blood urea nitrogen; Scr, serum creatinine.

Methods based on Cox proportional-hazards regression models were often accused of being unsuitable for dealing with non-linear models. As a result, we applied the Cox proportional hazards regression model with cubic spline functions and the smooth curve fitting (penalized spline method) to address the non-linearity between eGFR and pre-diabetes. After detecting non-linearity, we calculated the inflection point using a recursive algorithm and performed two-piecewise Cox proportional-hazards regression models on both sides of the inflection point. A log-likelihood ratio test was used to determine the most appropriate model for describing the risk associated with eGFR and pre-diabetes (27).

A stratified Cox proportional-hazards regression model was used across the various subgroups for subgroup analyses (gender, BMI, age, SBP, TG, DBP, drinking and smoking status, and family history of diabetes). Firstly, continuous variable age (<30, ≥30 to <40, ≥40 to <50, ≥50 to <60, ≥60 to <70, ≥70 years), BMI (<18.5, ≥18.5 to <24, ≥24 to 28, ≥28 kg/m^2^), SBP(<140, ≥140mmHg), DBP(<90, ≥90mmHg), TG(<1.7, ≥1.7mmol/L) were converted to a categorical variable based on the clinical cut point (28, 29). Secondly, we adjusted each stratification for all factors in addition to the stratification factor itself (SBP, gender, FPG, age, BMI, BUN, DBP, TG, ALT, HDL-c, AST, family history of diabetes, LDL-c, drinking and smoking status). Lastly, a likelihood ratio test for models with and without interaction terms was used to test for interactions (30, 31).

The number of participants with missing data of SBP, DBP, TC, TG, HDL-c, LDL-c, ALT, AST, BUN, smoking status, and drinking status were 13 (0.0075%), 14 (0.0081%), 2694 (1.55%), 2699 (1.56%), 74489 (42.98%), 74022 (42.71%), 976 (0.56%), 100474 (57.98%), 9503 (5.48%), 124705 (71.96%), and 124705 (71.96%), respectively. Multiple imputations were used to handle the missing data of covariants (32). The imputation model included BMI, age, SBP, HDL-c, gender, TC, DBP, ALT, TG, BUN, LDL-c, FPG, family history of diabetes, AST, drinking and smoking status. Missing data analysis procedures use missing-at-random (MAR) assumptions (33).

Sensitivity analyses were performed on our results to test their robustness. eGFR was transformed into a categorical variable according to the quartiles, and P for the trend was calculated to verify the results of eGFR as a continuous variable and explore the possibility of non-linearity. A decreased eGFR was defined as less than 90 ml/min/1.73m^2^ (16). Smoking and alcohol consumption are all related to an increased risk of type 2 diabetes mellitus (T2DM) (34). When exploring the association between eGFR and incident pre-diabetes in other sensitivity analyses, we excluded participants with a history of smoking and drinking, or eGFR<90 mL/min·1.73 m^2^. We also excluded drinking and smoking status from the multivariate model as sensitivity analysis. Smoking and drinking status did not have complete data in about 70% of the cases, and might not be useful as covariates to adjust in the model. The continuity covariate was also inserted into the equation (model IV) as a curve using a generalized additive model (GAM) to ensure the robustness of the results (35). Additionally, we calculated E-values to investigate the possibility of unmeasured confounding between eGFR and pre-diabetes risk (36). All results were written according to the STROBE statement (26).

Statistical analyses were performed using R (http://www.R-project.org, The R Foundation) and EmpowerStats (http://www.empowerstats.com, X&Y Solutions, Inc, Boston, MA). Statistical significance was determined by a P-value of <0.05 in all cases.

## Results

### Baseline characteristics of participants

The baseline characteristics of these included participants were listed in **Table 1**. The mean age was 40.95 ± 11.94 years, and 53.27% were male. The mean baseline eGFR was 111.40 ± 14.77 ml/min per 1.73 m^2^. During a median follow-up time of 3.0 years, 18333 (10.58%) people experienced pre-diabetes. We assigned the adults into subgroups using eGFR quartiles (<101.66, ≥101.66 to <113.55, ≥113.55 to <122.69, ≥122.69). When compared with the Q1 (<101.66) group, the values or proportions of HDL-c, females, never-smokers, never-drinkers, and family history of diabetes increased significantly in the Q4 (eGFR≥122.69) group. In contrast, the opposite results were detected in covariates in terms of SBP, age, LDL-c, BMI, FPG, TG, Scr, AST, DBP, TC, ALT, BUN, males, current or ever smokers, and current or ever drinkers.

**Figure 2:**
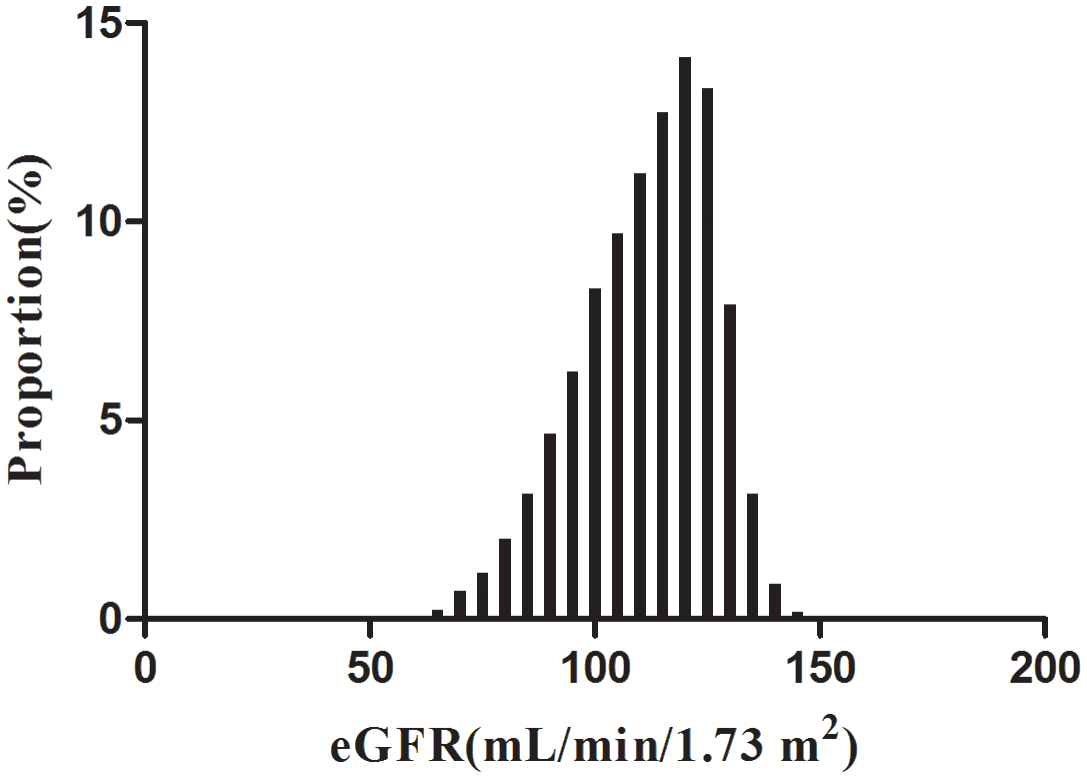
Distribution of eGFR. Figure 2. It presented a normal eGFR distribution while being in the range from 64.96 to 157.03 ml/min per 1.73 m^2^, with an average of 111.40 ml/min per 1.73 m^2^.

**Figure 3:**
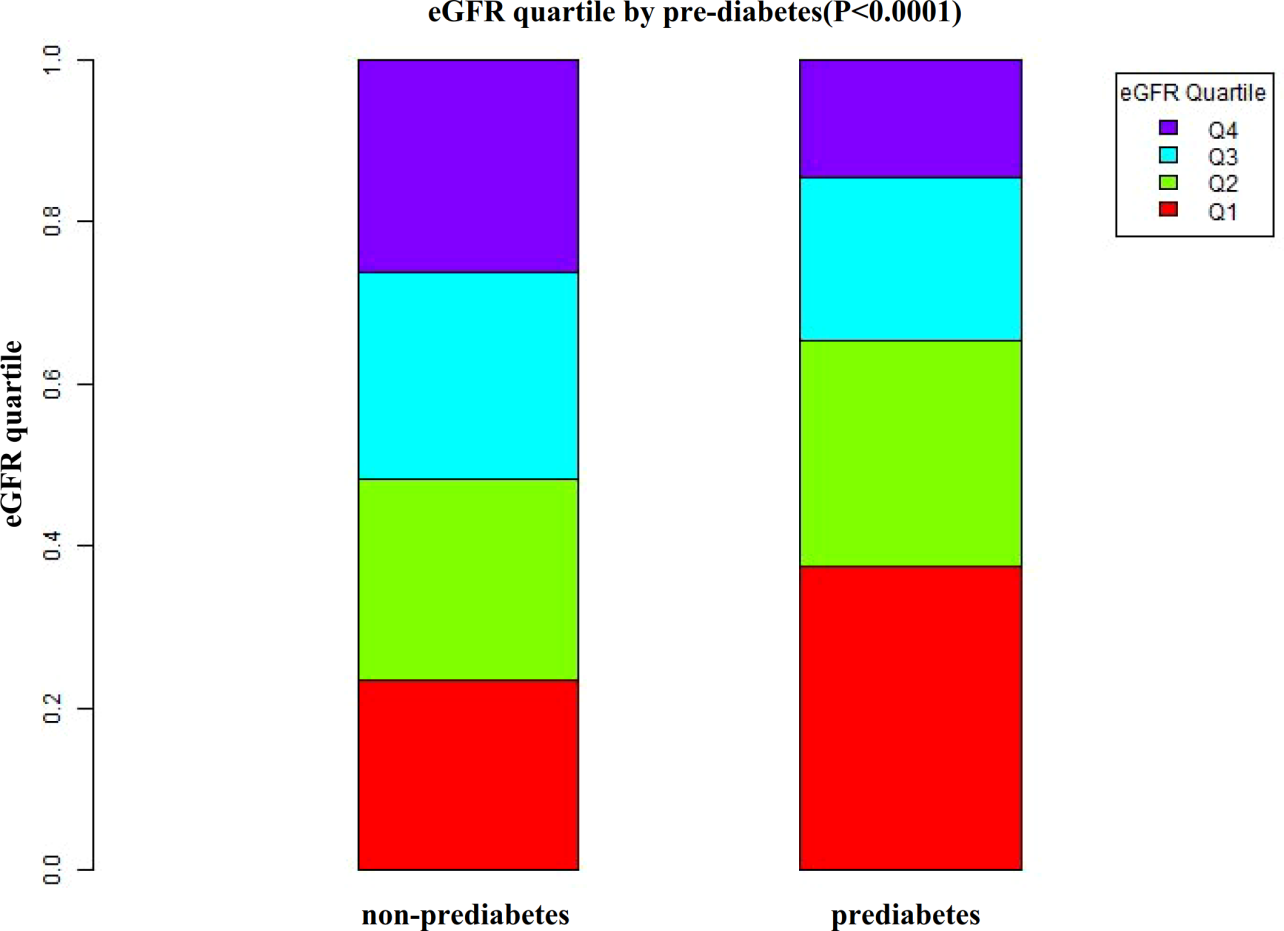
Data visualization of eGFR of all participants from the pre-diabetic and non-prediabetic groups. Figure 3 indicated that the level of eGFR in the pre-diabetic group was lower.

**Figure 4:**
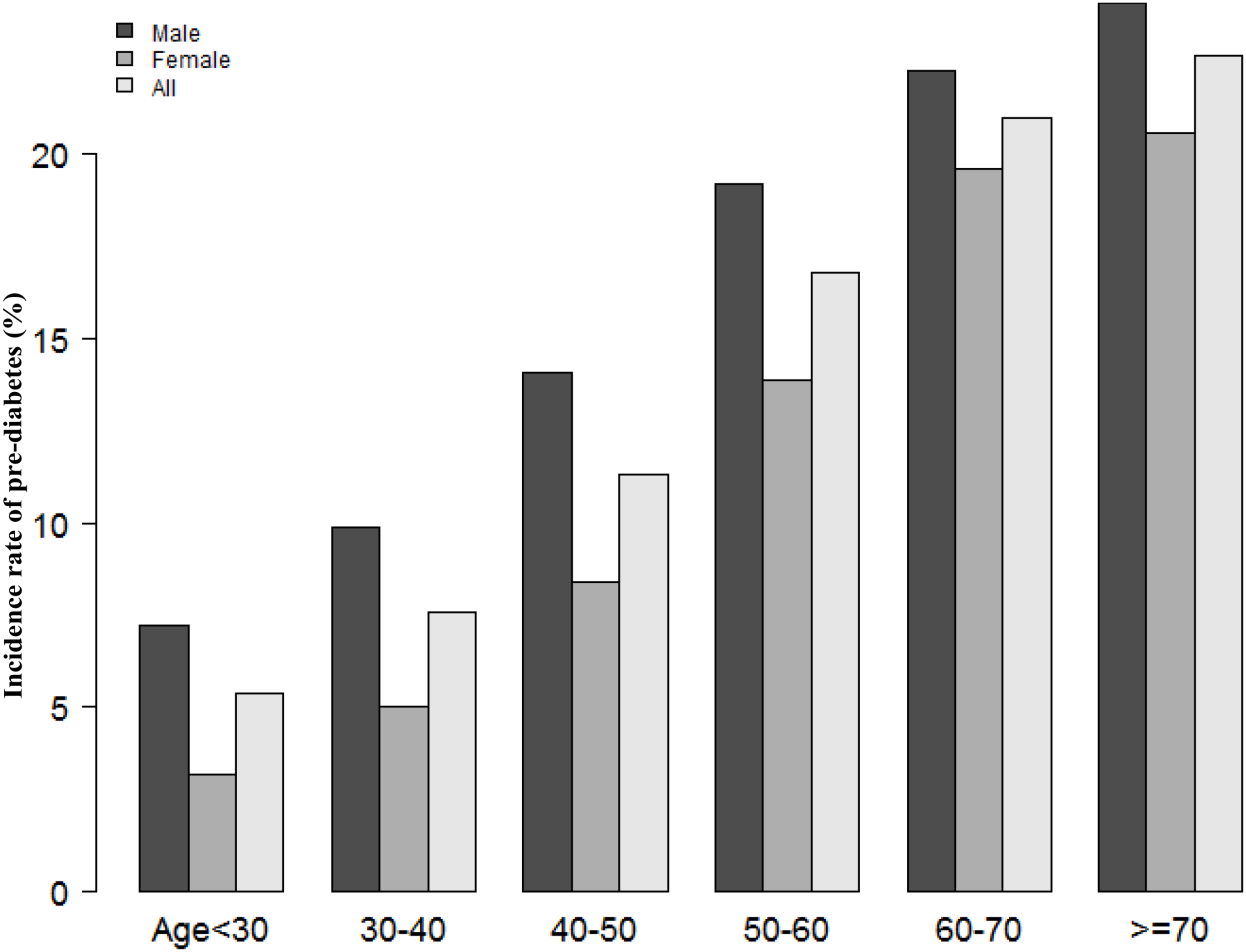
The pre-diabetes incidence rate of age stratification by 10 intervals. Figure 4 showed that in age stratification by 10 intervals, male subjects had a higher incidence of pre-diabetes than female subjects, no matter their age group. It also found that the incidence of pre-diabetes increased with age, both in males and females.

According to **Figure 2**, the eGFR levels had a normal distribution ranging from 64.96 to 157.03 ml/min per 1.73 m^2^, and the average was 111.40 ml/min per 1.73 m^2^. Based on whether participants developed pre-diabetes during the follow-up, participants were divided into two groups. As shown in **Figure 3**, the levels of eGFR in the non-prediabetic group were higher than those in the prediabetic group. Men were more likely to have pre-diabetes regardless of age group when age groups were stratified by 10 intervals (**Figure 4**). Furthermore, the incidence of pre-diabetes increased with age in both females and males.

### The incidence rate of pre-diabetes

**Figure 5:**
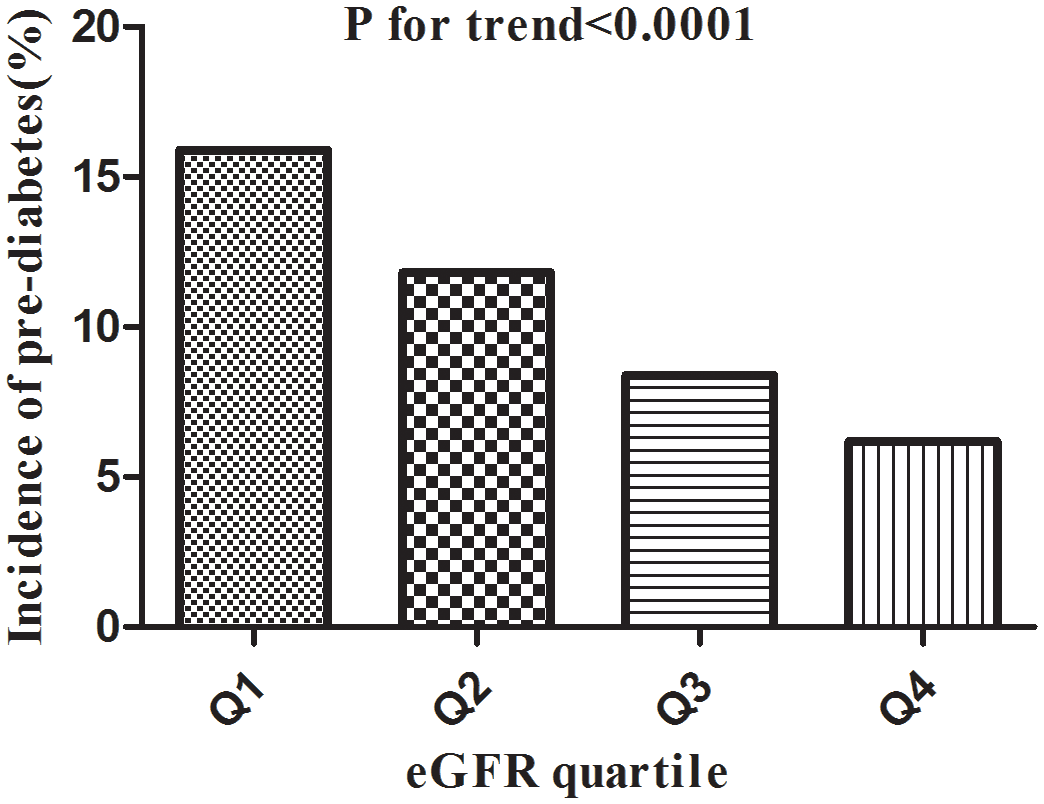
Incidence of pre-diabetes according to the quartiles of eGFR. Figure 5. Participants in the high eGFR group had a lower pre-diabetes incidence than the lowest eGFR group (p<0.0001 for trend).

**Table 2** revealed that 18333 (10.58%) participants developed pre-diabetes during a median follow-up time of 3.0 years. The total cumulative incidence rate of all persons was 3.37 per 100 person-years. In particular, the cumulative incidence of the four eGFR groups were 5.08, 3.73, 2.65, and 2.01 per 100 person-years, respectively. The incidence rate of total prediabetes and each eGFR group was 10.58% (10.43%-10.72%), 15.86% (15.51%-16.20%), 11.81% (11.51%-12.11%), 8.44% (8.18%-8.71%), and 6.21% (5.98%-6.43%), respectively. Participants with higher eGFR had lower incidence rates of pre-diabetes (P<0.0001 for trend) (**Figure 5**).

**Table 2:** Incidence rate of incident pre-diabetes.

eGFR	Participants (n)	Pre-diabetes events (n)	Incidence rate (95% CI) (%)	Cumulative incidence (Per 100 person-year)
**Total**	173301	18333	10.58 (10.43-10.72)	3.37
**Q1 (<101.66)**	43316	6868	15.86 (15.51-16.20)	5.08
**Q2 (101.66-113.55)**	43330	5117	11.81 (11.51-12.11)	3.73
**Q3 (113.55-122.69)**	43318	3658	8.44 (8.18-8.71)	2.65
**Q4 (≥122.69)**	43337	2690	6.21 (5.98-6.43)	2.01
**P for trend**			<0.001	

eGFR, estimated glomerular filtration rate (mL/min·1.73 m^2^).

### The results of univariate analyses using Cox proportional-hazards regression model

The univariate analysis showing that the factors in terms of the family history of diabetes were not connected with pre-diabetes. Still, age (HR=1.033, 95%CI 1.032-1.034), BMI (HR=1.123, 95%CI 1.119-1.128), SBP (HR=1.025, 95%CI 1.024-1.026), DBP (HR=1.029, 95%CI 1.028-1.030), FPG (HR=5.740, 95%CI 5.530-5.957), ALT (HR=1.003, 95%CI 1.003-1.004), AST (HR=1.006, 95%CI 1.005-1.006), TC (HR=1.219, 95%CI 1.201-1.238), TG (HR=1.199, 95%CI 1.190-1.208), LDL-c (HR=1.279, 95%CI 1.253-1.705), BUN (HR=1.144, 95%CI 1.131-1.158), Scr (HR=1.015, 95%CI 1.014-1.016), current (HR=1.387, 95%CI 1.338-1.437) or ever (HR=1.215, 95%CI 1.131-1.306) smokers, current (HR=1.778, 95%CI 1.626-1.944) or ever (HR=1.217, 95%CI 1.168-1.268) drinkers were positively correlated to prediabetes, and HDL-c (HR=0.795, 95%CI 0.759-0.834), females (HR=0.635, 95%CI 0.617-0.655), eGFR (HR=0.976, 95%CI 0.976-0.977) were negatively related with prediabetes (See **Table 3** for detail).

**Table 3:** The results of univariate analysis.

Variable	Statistics	HR (95%CI)	P value
**Age (years)**	40.947 ± 11.936	1.033 (1.032-1.034)	< 0.00001
**Gender**			
** Male**	92318 (53.270%)	Ref.	
** Female**	80983 (46.730%)	0.635 (0.617- 0.655)	< 0.00001
**BMI (Kg/m^2^)**	22.988 ± 3.259	1.123 (1.119- 1.128)	< 0.00001
**SBP (mmHg)**	117.671 ± 15.722	1.025 (1.024-1.026)	< 0.00001
**DBP (mmHg)**	73.469 ± 10.563	1.029 (1.028-1.030)	< 0.00001
**FPG (mmol/L)**	4.764 ± 0.488	5.740 (5.530-5.957)	< 0.00001
**TC (mmol/L)**	4.671 ± 0.883	1.219 (1.201-1.238)	< 0.00001
**TG (mmol/L)**	1.272 ± 0.937	1.199 (1.190-1.208)	< 0.00001
**HDL-c (mmol/L)**	1.373 ± 0.307	0.795 (0.759- 0.834)	< 0.00001
**LDL-c (mmol/L)**	2.691 ± 0.670	1.279 (1.253-1.305)	< 0.00001
**ALT (U/L)**	23.275 ± 21.646	1.003 (1.003-1.004)	< 0.00001
**AST (U/L)**	23.662 ± 12.074	1.006 (1.005-1.006)	< 0.00001
**eGFR (mL/min·1.73 m^2^)**	111.404 ± 14.768	0.976 (0.976-0.977)	< 0.00001
**BUN (mmol/L)**	4.598 ± 1.151	1.144 (1.131-1.158)	< 0.00001
**Scr (umol/L)**	69.386 ± 14.836	1.015 (1.014-1.016)	< 0.00001
**Smoking status**			
** Never smoker**	139662 (80.589%)	Ref.	
** Ever smoker**	6355 (3.667%)	1.215 (1.131-1.306)	< 0.00001
** Current smoker**	27284 (15.744%)	1.387 (1.338-1.437)	< 0.00001
**Drinking status**			
** Never drinker**	148940 (85.943%)	Ref.	
** Ever drinker**	21523 (12.419%)	1.217 (1.168-1.268)	< 0.00001
** Current drinker**	2838 (1.638%)	1.778 (1.626-1.944)	< 0.00001
**Family history of diabetes**			
** No**	169782 (97.969%)	Ref.	
** Yes**	3519 (2.031%)	1.039 (0.947-1.141)	0.41752

BMI, body mass index; FPG, fasting plasma glucose; DBP, diastolic blood pressure; TC, total cholesterol; SBP, systolic blood pressure; TG, triglyceride; ALT, alanine aminotransferase; LDL-c, low-density lipid cholesterol; AST, aspartate aminotransferase; HDL-c, high-density lipoprotein cholesterol; eGFR, estimated glomerular filtration rate; BUN, blood urea nitrogen; Scr, serum creatinine.

HR, Hazard ratios; CI, confidence interval; Ref, reference.

**Figure 6:**
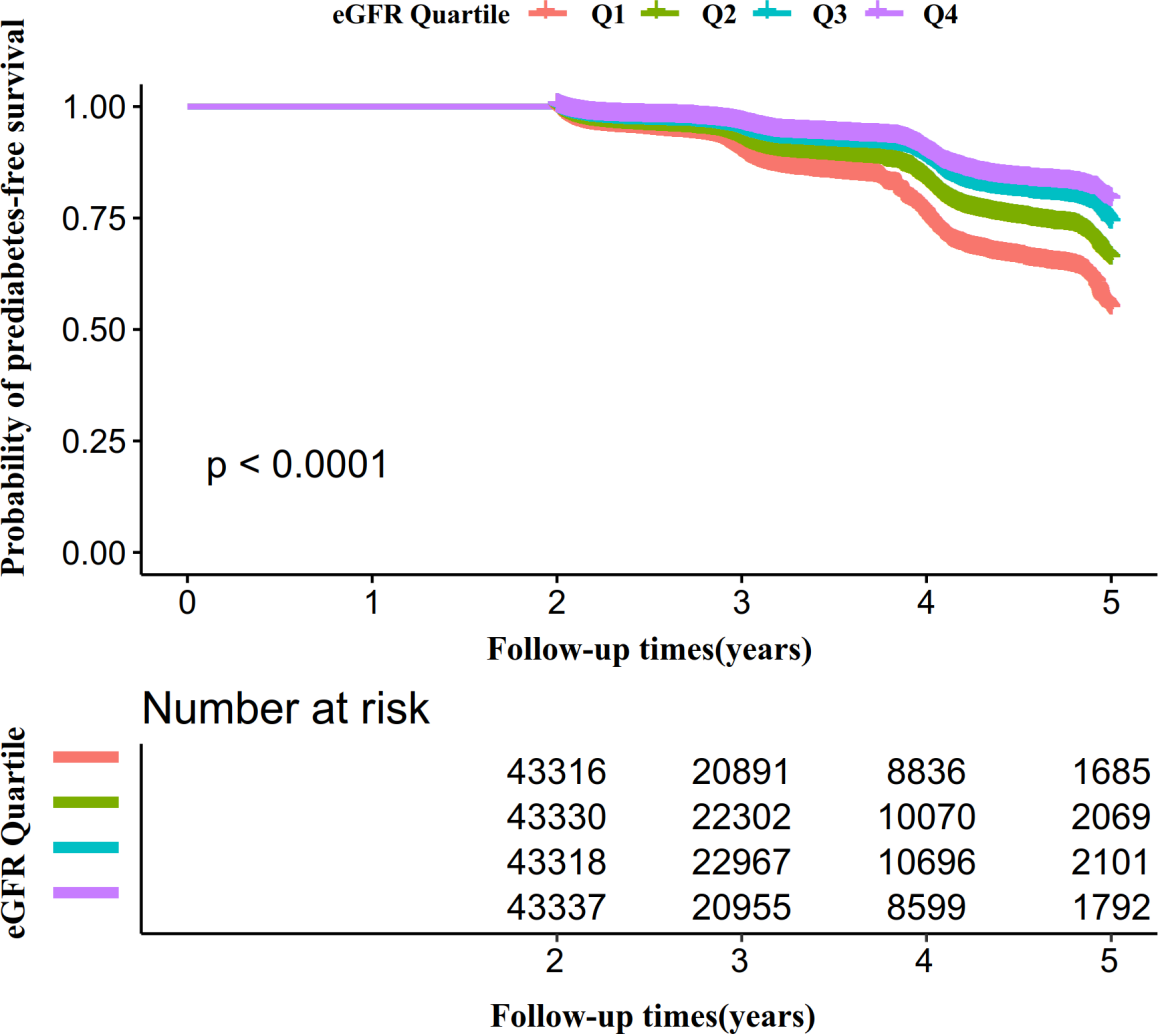
Kaplan–Meier event-free survival curve. Figure 6. Kaplan–Meier event-free survival curve. The probability of prediabetes-free survival differed significantly between the eGFR groups (log-rank test, P<0.0001). The probability of prediabetes-free survival gradually increased with increasing eGFR, suggesting that the group with the highest eGFR had the lowest risk of pre-diabetes.

Kaplan-Meier survival curves for prediabetes-free survival probability stratified by the eGFR group were shown in **Figure 6**. There were significant differences in the probability of prediabetes-free survival between the eGFR groups (log-rank test, P<0.0001). Prediabetes-free survival probabilities increased as eGFR increased, which indicated that those with the highest eGFR faced the lowest risk of pre-diabetes.

### Results from a multivariate Cox proportional-hazards regression model

To investigate the association between eGFR and pre-diabetes, the authors constructed three models using the Cox proportional-hazards regression model. In the unadjusted model (Model I), an increase of 1 mL/min·1.73 m^2^ of eGFR was related to a 2.4% decrease in the risk of pre-diabetes (HR=0.976, 95%CI 0.976-0.977). The results were statistically significant. In the minimally-adjusted model (Model II), when we only adjusted for demographic variables, each additional ml/min·1.73 m^2^ of eGFR decreased by 0.6% in the risk of pre-diabetes (HR=0.994, 95%CI 0.993-0.996). The findings on the link between eGFR and pre-diabetes obtained from the model were statistically significant. In the fully-adjusted model (Model III), each additional ml/min·1.73 m^2^ of eGFR was accompanied by a 0.7% decrease in pre-diabetes risk (HR=0.993, 95%CI 0.992-0.995). As shown by the distribution of confidence intervals, the relationship between eGFR and pre-diabetes obtained by the model was reliable.

### Sensitivity analysis

A series of sensitivity analyses were addressed to verify our findings’ robustness. We first transform eGFR into a categorical variable (based on quartile) and then incorporate the categorical-transformed eGFR into our model. The results showed that the trends in effect sizes between groups were not wholly equivalent after transforming eGFR into a categorical variable, indicating a possible non-linear relationship between eGFR and pre-diabetes.

In addition, to include the continuity covariate as a curve in the equation, we used a GAM. The result of Model IV in **Table 4** showed this generally remained consistent with the fully adjusted model (HR=0.994, 95%CI: 0.992-0.995, P<0.0001). Besides, E-values were generated to test sensitivity to unmeasured confounding. The E-value was 1.09. The E-value was greater than the relative risk of unmeasured confounders and pre-diabetes, suggesting unmeasured or unknown confounders had little effect on the relationship between eGFR and incident pre-diabetes.

**Table 4:** Relationship between eGFR and the incident pre-diabetes in different models.

Exposure	Model I (HR,95%CI,P)	Model II (HR,95%CI,P)	Model III (HR,95%CI,P)	Model IV (HR,95%CI, P)
**eGFR**	0.976 (0.976-0.977) <0.0001	0.994 (0.993-0.996) < 0.0001	0.993 (0.992-0.995) < 0.0001	0.994 (0.992-0.995) < 0.0001
**eGFR Quartile**				
** Q1**	Ref.	Ref.	Ref.	Ref.
** Q2**	0.695 (0.670-0.720) < 0.0001	0.925 (0.889-0.962) < 0.0001	0.892 (0.858-0.928) < 0.0001	0.885 (0.850-0.921) < 0.0001
** Q3**	0.484 (0.465-0.504) < 0.0001	0.807 (0.769-0.846) < 0.0001	0.788 (0.751-0.827) < 0.0001	0.814 (0.775-0.855) < 0.0001
** Q4**	0.398 (0.381-0.416) < 0.0001	0.818 (0.773-0.866) < 0.0001	0.795 (0.751-0.842) < 0.0001	0.807 (0.761-0.857) < 0.0001
**P for trend**	< 0.0001	< 0.0001	< 0.0001	< 0.0001

Model I: we did not adjust other covariates.

Model II: we adjust age, gender, BMI, SBP, DBP, family history of diabetes, smoking and drinking status.

Model III: we adjust age, gender, BMI, SBP, DBP, FPG, BUN, TG, HDL-c, LDL-c, ALT, AST, family history of diabetes, smoking and drinking status.

Model IV: we adjusted age(smooth), gender, BMI(smooth), SBP(smooth), DBP(smooth), FPG(smooth), BUN(smooth), TG(smooth), HDL-c(smooth), LDL-c(smooth), ALT(smooth), AST(smooth), family history of diabetes, smoking and drinking status.

HR, Hazard ratios; CI: confidence, Ref: reference; eGFR, estimated glomerular filtration rate(mL/min·1.73 m^2^).

Furthermore, the authors excluded participants with eGFR<90mL/min·1.73 m^2^ (N=156,968) for the sensitivity analysis. A negative association of eGFR with pre-diabetes risk was also observed after adjusting for confounding factors (HR=0.994, 95%CI:0.992-0.996) (**Table 5**). We also excluded participants with a history of smoking. The results showed that after adjusting SBP, gender, FPG, age, BMI, BUN, DBP, TG, ALT, HDL-c, AST, family history of diabetes, LDL-c, drinking status, eGFR was still negatively associated with prediabetes (HR=0.992, 95% CI:0.991- 0.994) (**Table 5**). For sensitivity analyses, we also excluded persons with a history of drinking. We still got similar results (HR=0.994, 95% CI:0.992-0.995).

**Table 5:** Relationship between eGFR and pre-diabetes in different sensitivity analyses.

Exposure	Model a (HR,95%CI, P)	Model b (HR,95%CI, P)	Model c (HR,95%CI, P)	Model d (HR,95%CI, P)
**eGFR**	0.994 (0.992-0.996) < 0.0001	0.992 (0.991-0.994) < 0.0001	0.994 (0.992-0.995) < 0.0001	0.993 (0.992-0.995) < 0.0001
**eGFR (Quartile)**				
** Q1**	Ref.	Ref.	Ref.	Ref.
** Q2**	0.924 (0.884-0.965) 0.0004	0.873 (0.833-0.914) < 0.0001	0.900 (0.862-0.940) < 0.0001	0.893 (0.858-0.928) < 0.0001
** Q3**	0.823 (0.780-0.867) < 0.0001	0.761 (0.720-0.804) < 0.0001	0.803 (0.762-0.847) < 0.0001	0.788 (0.751-0.827) < 0.0001
** Q4**	0.837 (0.786-0.891) < 0.0001	0.769 (0.721-0.822) < 0.0001	0.804 (0.755-0.857) < 0.0001	0.796 (0.752-0.844) < 0.0001
**P for trend**	< 0.0001	< 0.0001	< 0.0001	< 0.0001

Model a was sensitivity analysis in participants without eGFR<90mL/min·1.73 m^2^ (N=156,968).We adjusted age, gender, BMI, SBP, DBP, FPG, BUN, TG, HDL-c, LDL-c, ALT, AST, family history of diabetes, smoking and drinking status.

Model b was a sensitivity analysis performed on never smoker participants (N=139,662). We adjusted adjusted age, gender, BMI, SBP, DBP, FPG, BUN, TG, HDL-c, LDL-c, ALT, AST, family history of diabetes, and drinking status.

Model c was a sensitivity analysis performed on never drinker participants (N=148,940). We adjusted age, gender, BMI, SBP, DBP, FPG, BUN, TG, HDL-c, LDL-c, ALT, AST, family history of diabetes, and smoking status.

Model d was sensitivity analysis in participants without adjusting smoking and drinking status (N=173,301). We adjusted age, gender, BMI, SBP, DBP, FPG, BUN, TG, HDL-c, LDL-c, ALT, AST, family history of diabetes.

HR, Hazard ratios; CI: confidence, Ref: reference; eGFR, estimated glomerular filtration rate(mL/min·1.73 m^2^).

Due to the fact that smoking and alcohol status had about 70 percent of missing data, these data might not be suitable as covariates. We excluded drinking and smoking status from the multivariate model in other sensitivity analyses. It was still similar to the previous results (HR=0.993, 95% CI:0.992-0.995). The results obtained from sensitivity analysis indicated the well-robustness of our findings.

### The non-linearity addressed by Cox proportional hazards regression model with cubic spline functions

**Figure 7:**
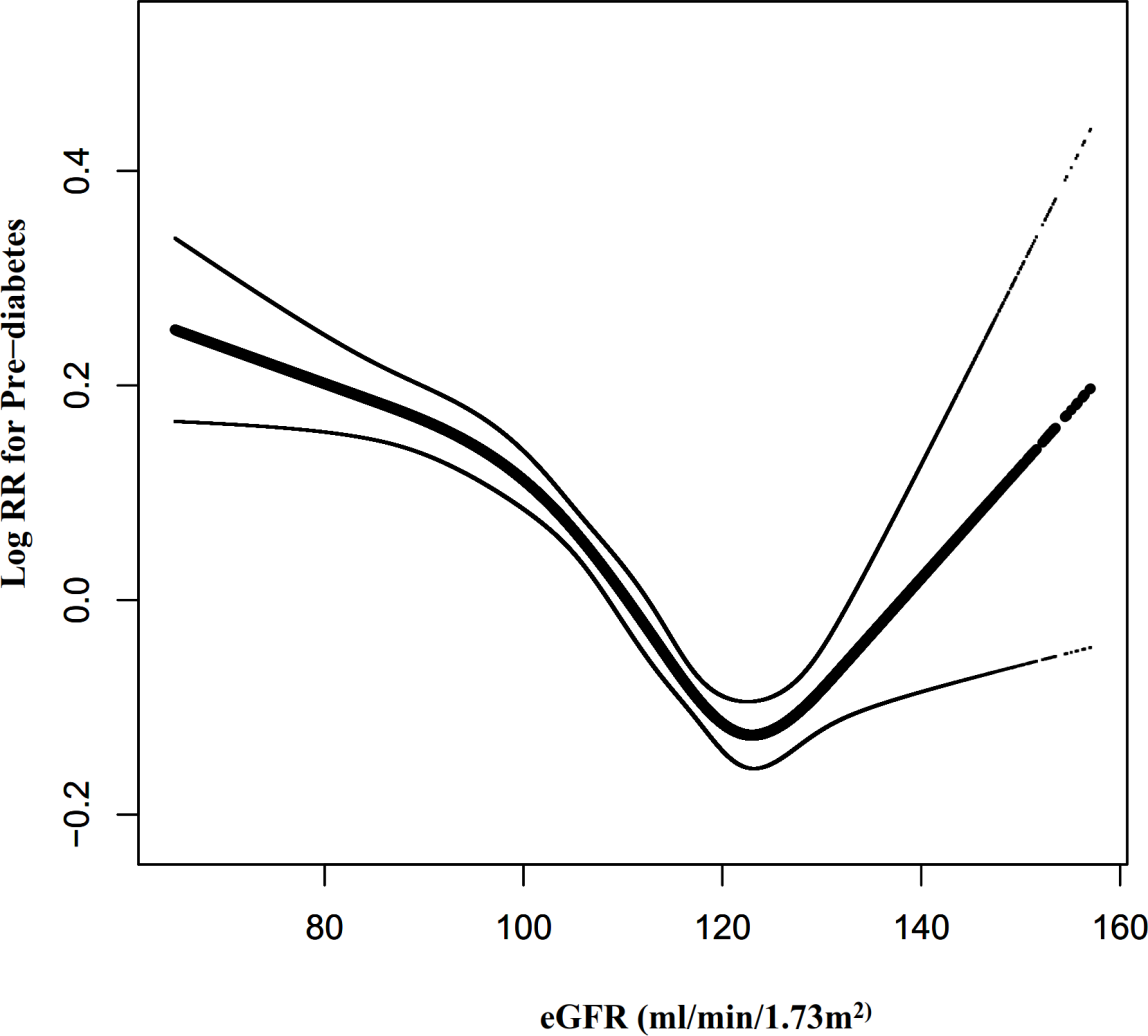
The non-linear relationship between eGFR and the risk of prediabetes. Figure 7. We used a Cox proportional hazards regression model with cubic spline functions to evaluate the relationship between eGFR and pre-diabetes risk. The relationship between eGFR and pre-diabetes showed a U-shaped curve with an inflection point of 129.79mL/min·1.73 m^2^.

Cox proportional hazards modeling with cubic spline functions and smooth curve fitting revealed that eGFR and pre-diabetes follow a U-shaped relationship (**Figure 7**). On the basis of the sensitivity analysis, we fitted data by a standard Cox proportional-hazards regression model and selected the best-fitting model by calculating log-likelihood ratios. We found that the P for the log-likelihood ratio test was less than 0.05. By recursive algorithm, we first obtained the inflection point was 129.793 ml/min·1.73 m^2^ and then calculated HR and CI on both sides of the inflection point using two-piecewise Cox proportional-hazards regression models. At the inflection point on the left side, the HR and 95%CI were 0.993(0.991-0.994), respectively. At the inflection point on the right side, they were 1.023(1.010- 1.037), respectively (**Table 6**).

**Table 6:** The result of the two-piecewise Cox regression model.

Incident pre-diabetes	Model* (HR,95%CI, P)	Model#(HR,95%CI, P)
**Fitting model by standard Cox regression**	0.993 (0.992-0.995) < 0.0001	0.994 (0.992-0.996) < 0.0001
**Fitting model by two-piecewise Cox regression**		
** Inflection point of eGFR**	129.793	124.122
** ≤Inflection point**	0.993 (0.991-0.994) < 0.0001	0.991 (0.989-0.993) < 0.0001
** >Inflection point**	1.023 (1.010-1.037) < 0.0001	1.011 (1.004-1.018) 0.0036
**P for log-likelihood ratio test**	< 0.001	< 0.001

Model *: Analysis among all participants; Model II#: Sensitivity analysis in participants without eGFR<90mL/min·1.73 m^2^ (N=156,968).

We adjusted age, gender, BMI, SBP, DBP, FPG, BUN, TG, HDL-c, LDL-c, ALT, AST, family history of diabetes, smoking and drinking status.

HR, Hazard ratios; CI: confidence, Ref: reference; eGFR, estimated glomerular filtration rate(mL/min·1.73 m2).

**Figure 8:**
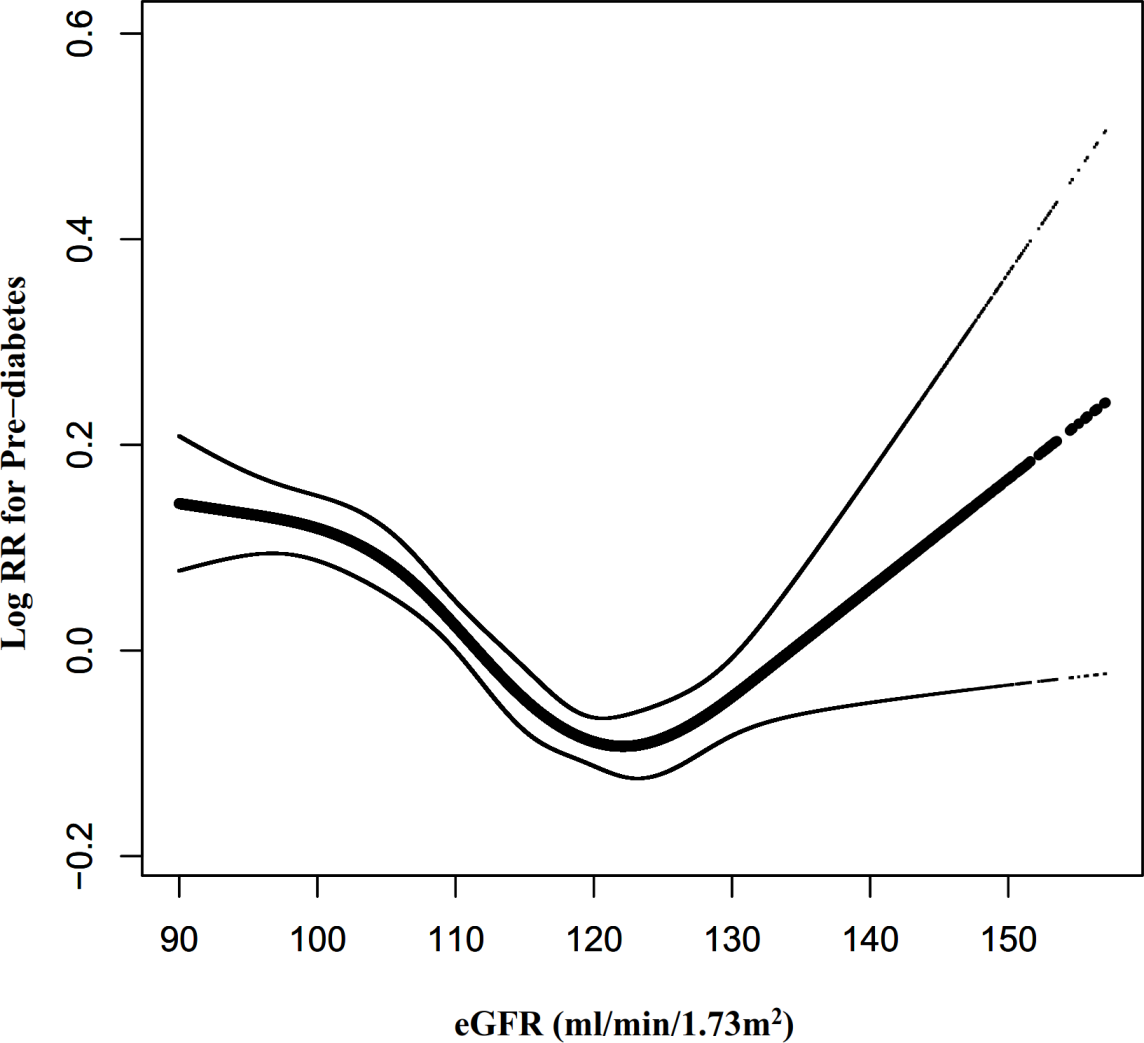
The non-linear relationship between eGFR and pre-diabetes risk in participants with eGFR≥90 mL/min·1.73 m2. Figure 8. We also used a Cox proportional hazards regression model with cubic spline functions to evaluate the relationship between eGFR and pre-diabetes risk in participants with eGFR≥90 mL/min·1.73 m^2^. The relationship between eGFR and pre-diabetes also showed a U-shaped curve with an inflection point of 124.12mL/min·1.73 m^2^.

We also excluded participants with eGFR<90mL/min·1.73 m^2^ for sensitivity analysis when exploring the non-linear relationship between eGFR and incident pre-diabetes. The results showed that the U-shaped relationship between eGFR and pre-diabetes still existed (**Figure 8**). Specifically, the inflection point of eGFR was 124.122 ml/min·1.73 m^2^. On the left and right sides of the inflection point, the HR and 95%CI were 0.991 (0.989-0.993) and 1.011 (1.004-1.018), respectively (**Table 6**).

### The results of subgroup analyses

In all of the prespecified or exploratory subgroups evaluated (**Table 7**), there was no significant interaction in age, BMI, SBP, DBP, gender, TG, family history of diabetes, drinking status, or SBP. In contrast, significant interactions were detected in variables such as age and smoking status.

**Table 7:** Effect size of eGFR on incident pre-diabetes in prespecified and exploratory subgroups.

Characteristic	No of participants	HR (95%CI)	P value	P for interacion
**Age(years)**				0.0019
** 20 to <30**	25242	0.989 (0.984-0.993)	< 0.0001	
** 30 to <40**	72109	0.996 (0.994-0.999)	0.0026	
** 40 to <50**	37203	0.990 (0.988-0.993)	< 0.0001	
** 50 to <60**	22243	0.991 (0.989-0.994)	< 0.0001	
** 60 to <70**	12249	0.992 (0.988-0.996)	< 0.0001	
** ≥70**	4255	0.996 (0.989-1.003)	0.2626	
**Gender**				0.4950
** Male**	92318	0.994 (0.992-0.995)	< 0.0001	
** Female**	80983	0.993 (0.990-0.995)	< 0.0001	
**BMI(kg/m^2^)**				0.0917
** <18.5**	10871	0.987 (0.979-0.996)	0.0031	
** ≥18.5, <24**	99904	0.994 (0.992-0.997)	< 0.0001	
** ≥24, <28**	49962	0.993 (0.991-0.995)	< 0.0001	
** ≥28**	12564	0.990 (0.987-0.994)	< 0.0001	
**Smoking status**				0.0060
** Never smoker**	139662	0.992 (0.991-0.994)	< 0.0001	
** Ever smoker**	6355	0.993 (0.988-0.999)	0.0312	
**Current smoker**	27284	0.997 (0.994-1.000)	0.0708	
**Drinking status**				0.7025
** Never drinker**	148940	0.994 (0.992-0.995)	< 0.0001	
** Ever drinker**	21523	0.992 (0.989-0.995)	< 0.0001	
** Current drinker**	2838	0.991 (0.983, 1.000)	0.0379	
**Family history of diabetes**				0.4926
** No**	169782	0.993 (0.992, 0.994)	< 0.0001	
** Yes**	3519	0.995 (0.989, 1.002)	0.5208	
**SBP(mmHg)**	159153			0.3043
** <140**		0.994 (0.992, 0.995)	< 0.0001	
** ≥140**	14148	0.992 (0.989, 0.995)	< 0.0001	
**DBP(mmHg)**	161318			0.3049
** <90**	11983	0.993 (0.992, 0.995)	< 0.0001	
** ≥90**		0.991 (0.988, 0.995)	< 0.0001	
**TG(mmol/L)**				0.1170
** <1.7**	137654	0.993 (0.991, 0.994)	< 0.0001	
** ≥1.7**	35647	0.995 (0.993, 0.997)	< 0.0001	

Note 1: Above model adjusted for age, gender, BMI, SBP, DBP, FPG, BUN, TG, HDL-c, LDL-c, ALT, AST, family history of diabetes, smoking and drinking status.

Note 2: In each case, the model is not adjusted for the stratification variable.

Specifically, a stronger association between eGFR and prediabetes was observed in the participants who were younger than 30 years (HR=0.989,95%CI:0.984-0.993) and 40-70 years (HR=0.990,95%CI:0.988-0.993; HR=0.991,95%CI:0.989-0.994; HR=0.992,95%CI:0.988-0.996), as well as among those who had never smoked (HR=0.992,95%CI:0.991-0.994). In contrast, the association of eGFR with the risk of incident pre-diabetes was attenuated among participants who were 30-40 years of age (HR=0.996,95%CI:0.994-0.999) and 70 years of age or older (HR=0.996,95%CI:0.989-1.003), and among those who currently smoked (HR=0.997,95%CI:0.994-1.000).

## Discussion

Our retrospective cohort study was designed to examine the link between eGFR and pre-diabetes in the Chinese community. We found that the increase in eGFR was connected with a significantly decreased risk of pre-diabetes. Moreover, a U-shaped curve was also identified, and significant associations between eGFR and pre-diabetes were seen on both sides of the inflection point. In addition, as potential moderators of the relationship between eGFR and pre-diabetes, age and smoking status were found to be significant, as significantly stronger associations were observed in age (20–30, 40–70) and never smokers.

A recent study by Zou et al. identified 12.31% of participants with new-onset pre-diabetes over a median observation period of 3.1 years (37). Our study found that pre-diabetes incidence was 10.58% after a median follow-up of 3.0 years in the population with physical examination in the Chinese community. The incidence of pre-diabetes was slightly lower in our study population. By comparing people in both cohorts, those without lipid parameters were excluded from the study by Zou et al. (37). The final sample size was 100309, and their study had a maximum follow-up time of about 6 years. In our study, however, those without lipid indicators were not excluded, and the total sample size was 173301, with a maximum follow-up time of 5 years. This might account for our study population’s slightly lower incidence of pre-diabetes.

In general, pre-diabetes is associated with microalbuminuria, impaired kidney function, and chronic kidney disease, according to a German study (38). According to another Chinese cohort study, pre-diabetes is positively associated with renal dysfunction (OR=1.72, 95% CI 1.11-22.38) (39). By Cox proportional hazards regression model analysis, our study found that the decline in eGFR was strongly associated with an increased risk of pre-diabetes, which was consistent with previous findings. In addition, according to another study, fasting blood glucose levels but not 2h post-load levels or HbA1c was associated with a mild decline in eGFR among healthy Chinese adults. A mild reduction in eGFR was defined as 60-90 mL/min/1.73 m^2^ (16). Concurrently, our sensitivity analysis found that the relationship remained stable among participants who never smoked or drank, and among those who did not have an eGFR<90 mL/min/1.73 m^2^. We also found that the negative association between eGFR and pre-diabetes remained stable when the multiple regression equation did not adjust smoking and drinking status. The efforts as mentioned above have confirmed the relationship’s stability between eGFR and pre-diabetes risk. The results provided a reference for clinical intervention in eGFR levels to reduce the risk of pre-diabetes.

However, in a large Japanese cohort study, researchers found that pre-diabetes was independently related to the development of proteinuria (OR=1.233; 95%CI1.170-1.301), whereas pre-diabetes was not associated with the decline in eGFR (OR=0.981; 95% CI 0. 947-1.017). eGFR decline was defined as eGFR<90 mL/min/1.73 m^2^ (40). The reasons why their findings are inconsistent with ours may include the following (1): The study population was different. Their study mainly focused on the Japanese, while ours focused on Chinese people. (2) The study design and the regression analysis methods used to explore the relationship between renal function and pre-diabetes were different. They explored the association between pre-diabetes and renal function decline (eGFR< 60ml/min/1.73m^2^) by logistic regression analysis. In contrast, our study analyzed the association by the Cox proportional hazards model. In addition, they did not examine the non-linear relationship between eGFR and pre-diabetes. (3) Compared with our research, their study did not consider the effect of BUN, FPG, ALT, AST, drinking status, and family history of diabetes on the relationship between eGFR and pre-diabetes when adjusting covariates. However, previous studies have identified these variables as factors associated with pre-diabetes or eGFR (41–44). (4) This might be related to different renal functions. Several studies suggested that the association of eGFR and insulin resistance (IR) differs between CKD stages (45, 46). It has also been shown that patients with impaired glucose tolerance and newly diagnosed pre-diabetes have an increased risk of glomerular hyperfiltration (17). The above studies also illustrated that the relationship between different eGFR levels and pre-diabetes might be different, so it is important to explore the non-linear relationship between eGFR levels and the risk of pre-diabetes.

Furthermore, to the best of our knowledge, the present study observed a non-linear relationship between eGFR and pre-diabetes risk for the first time. The current study used a two-piecewise Cox proportional hazards regression model to clarify a U-shaped relationship between eGFR and pre-diabetes risk. The inflection point of eGFR was 129.793 ml/min/1.73m^2^ after adjusting for confounders. It showed that when eGFR was below 130 ml/min/1.73m^2^, a 1 unit decrease in the eGFR level was associated with a 0.7% greater adjusted HR of pre-diabetes risk (HR=0.993, 95%CI: 0.991-0.994). However, when eGFR was above 130 ml/min/1.73m^2^, a 1 unit increase in eGFR level was associated with a 2.3% greater adjusted HR of the risk of pre-diabetes (HR=1.023, 95%CI: 1.010-1.037). The results suggested that the HR of incident pre-diabetes was lowest when the eGFR was around 130ml/min/1.73m^2^. Multiple studies have confirmed that pre-diabetes is strongly associated with decreased eGFR and the risk of CKD (15, 17, 38, 39). It has also been shown that a decrease in eGFR is associated with an increase in IR (46). This would explain our findings that when eGFR is less than 130 ml/min/1.73m^2^, declining eGFR is associated with an increased risk of developing pre-diabetes in the future. However, it has also been shown that pre-diabetes is strongly related to glomerular hyperfiltration (17, 47–49). Glomerular hyperfiltration has been associated with the early stages of nephropathy that evolved into CKD (50). In addition, insulin resistance is associated with renal dysfunction, playing an essential role in glomerular hyperfiltration, endothelial dysfunction, and increased vascular permeability (51). This would explain the finding in our study that when eGFR was > 130 ml/min/1.73m^2^, the risk of incident pre-diabetes increased with increasing eGFR. Furthermore, Lorenzo et al. found that individuals in the upper and lower ranges of GFR were more likely to develop diabetes in the future (52). It is a reasonable approach to prevent prediabetes with reference to eGFR levels in clinical practice. This study provides a reference for preventing pre-diabetes in people with different renal function statuses in the future. It is important to be clinically alert to the increased risk of prediabetes in patients with decreased renal function on the one hand, and also to the early renal damage such as glomerular hyperperfusion in patients with prediabetes. Therefore, this assay has excellent clinical value. The findings of this research should be conducive to future studies on establishing a predictive model of pre-diabetes risk.

In subgroup analysis, we found that smoking status could be the potential effect modifier to modify the relationship between eGFR and pre-diabetes risk. Stronger associations are observed in the population who have never smoked. Studies have shown that smoking is associated with insulin resistance (53, 54). In our research, smoking was also associated with an increased risk of pre-diabetes. Therefore, it is not surprising that the association of eGFR with pre-diabetes in those currently smoking cigarettes is weakened by the influence of smoking. Since smoking status could modify the relationship between eGFR and pre-diabetes, it is clinically possible to reduce pre-diabetes’ risk by altering the association strength between the eGFR and pre-diabetes by controlling or reducing smoking.

There are several strengths to our study, and we listed them below. (1) A strength of our research is that the total sample size was relatively large. (2) To the best of our knowledge, this is the first time Chinese people have been used as a research population to explore the relationship between eGFR and pre-diabetes. (3) This study explores non-linearity and explains them further. This is a very significant improvement over previous studies. (4) We used multiple imputations to handle missing data in this study. Multiple imputations could maximize statistical power and minimize potential bias caused by covariate information missing. (5) Since this is an observational study, it is vulnerable to potential confounding. We minimized residual confounding by using strict statistical adjustment. (6) Throughout this study, a series of sensitivity analyses were conducted to ensure the reliability of the results (conversion of target-independent variable form, subgroup analysis, using a GAM to insert the continuity covariate into the equation as a curve, calculating E-values to explore the potential for unmeasured confounding, and reanalyzing the association between eGFR and pre-diabetes among participants who never smoked or drank, and among those who did not have an eGFR<90 mL/min/1.73 m^2^). This makes our results more reliable.

Our research has the following shortcomings and needs attention: First, the design of this study is an observational study, so we cannot get the exact causal relationship because of the nature of the observational study design. Second, as in all observational studies, even though known potential confounding factors, such as BMI, age, FPG, and BUN, were controlled, there might have been still uncontrolled or unmeasured confounders, such as physical activity. In spite of this, the authors calculated the E-value in order to quantify the impact of unmeasured confounders and determined that they were unlikely to explain the results. In the future, we can consider designing our studies or collaborating with other researchers to collect as many variables as possible, including information on physical activity. Third, time to event is impossible to know in this study as the state of prediabetes is mostly without any symptoms, and patients could not tell when they have had it; therefore, the visit will also influence the time to event. Using logistic regression models, we re-analyzed the association between eGFR and prediabetes (**Table S2**). The results showed that the relationship between eGFR and prediabetes analyzed by logistic regression was consistent with the Cox proportional risk model results. Fourth, the study only measured eGFR at baseline and did not account for changes in eGFR over time. In addition, taking ACE inhibitors or ARBs may affect eGFR levels and their subsequent changes, and other medications may also affect eGFR and the risk of prediabetes. Therefore it is necessary to exclude participants using ACE inhibitors or ARBs at baseline, as well as other drugs that affect eGFR and risk of prediabetes. Finally, the researchers in this study only diagnosed pre-diabetes in the participants with impaired fasting glucose levels during follow-up, and the information on the OGTT, HbA1c, and multiple measurements of FPG was missing, which might lead to a missed diagnosis (55). As a result, future studies should include as many variables as possible, including information on the changes in renal function during the study period, the use of medications, OGTT, HbA1c, and multiple measurements of FPG. And we also need to design the questionnaire carefully.

## Conclusion

This study demonstrates a negative and U-shaped relationship between eGFR and pre-diabetes risk in the Chinese community-based population. The HR of incident pre-diabetes was lowest when the eGFR was around 130ml/min/1.73m^2^. This study provides a reference for preventing pre-diabetes in people with different renal function statuses in the future. Protecting renal function may be a new therapeutic direction to reduce pre-diabetes risk.

## Data availability statement

The datasets presented in this study can be found in online repositories. The names of the repository/repositories and accession number(s) can be found in the article/**Supplementary Material**.

## Ethics statement

The studies involving human participants were reviewed and approved by the Rich Healthcare Group Review Board. Written informed consent for participation was not required for this study in accordance with the national legislation and the institutional requirements.

## Author contributions

XW, CH, YH, YL, and HH conceived the research, drafted the manuscript, and did the statistical analysis. HH revised the manuscript and designed the study. All authors contributed to the article and approved the submitted version.

## Funding

This study was supported by the Discipline Construction Ability Enhancement Project of the Shenzhen Municipal Health Commission (SZXJ2017031) and the Shenzhen Key Medical Discipline Construction Fund (SZXK009).

## Acknowledgments

As this is a secondary analysis, the data and method description are mainly derived from the following research: Chen Y, Zhang XP, Yuan J, Cai B, Wang XL, Wu XL, Zhang YH, Zhang XY, Yin T, Zhu XH, Gu YJ, Cui SW, Lu ZQ, Li XY. Association of body mass index and age with incident diabetes in Chinese adults: a population-based cohort study. BMJ Open. 2018 Sep 28;8(9):e021768. doi: 10.1136/bmjopen-2018-021768. All authors of this study are grateful for their contributions.

## Conflict of interest

The authors declare that the research was conducted in the absence of any commercial or financial relationships that could be construed as a potential conflict of interest.

## Publisher’s note

All claims expressed in this article are solely those of the authors and do not necessarily represent those of their affiliated organizations, or those of the publisher, the editors and the reviewers. Any product that may be evaluated in this article, or claim that may be made by its manufacturer, is not guaranteed or endorsed by the publisher.

## Abbreviations

ADA, American Diabetes Association; CKD-EPI, Chronic Kidney Disease Epidemiology Collaboration; SBP, Systolic blood pressure; T2DM, Type 2 diabetes mellitus; DM, Diabetes mellitus; Scr, Serum creatinine; HbA1c, Glycated hemoglobin; ALT, Alanine aminotransferase; BMI, Body mass index; TG, Triglyceride; DBP, Diastolic blood pressure; eGFR, Estimated glomerular filtration rate; LDL-c, Low-density lipid cholesterol; VIF, Variance inflation factor; HDL-c, High-density lipoprotein cholesterol; CKD, Chronic kidney disease; AST, Aspartate aminotransferase; FPG, Fasting plasma glucose; BUN, Blood urea nitrogen; GAM, Generalized additive models; TC, Total cholesterol; HR, hazard ratios; Ref, Reference; CI, Confidence intervals; MAR, missing-at-random; IFG, Impaired fasting glucose; IR, Insulin resistance; SD, Standard deviation.
